# Toxic shock syndrome and pyomyositis: about an unusual case

**DOI:** 10.11604/pamj.2020.36.63.23237

**Published:** 2020-06-03

**Authors:** Mouad EL Mouhtadi, Karima EL Fakiri, Noureddine Rada, Ghizlane Draiss, Youssef Mouaffak, Said Younous, Mohammed Bouskraoui

**Affiliations:** 1 Pediatric A Department, University Hospital Med VI, Marrakesh, Morocco,; 2 Pediatric Intensive Care Department, University Hospital Med VI, Marrakesh, Morocco

**Keywords:** Pyomyositis, magnetic resonance imaging, toxic shock syndrome

## Abstract

Pyomyositis is a pyogenic infection of skeletal muscle with abscess formation. It is a rare disease with nonspecific symptoms which requires a rapid diagnosis and treatment. Magnetic resonance imaging is considered the gold standard for early diagnosis and to rule out other etiologies. This article reports an atypical presentation of pyomyositis revealed by a toxic staphylococcal shock syndrome in an 8-year-old boy.

## Introduction

Pyomyositis is a primary infection of the skeletal muscle usually with abscess formation. It is a rare condition initially described in 1885, occurring classically in tropical countries. Its pathogenesis is not yet fully understood, however, it is thought to be induced by bacteremia where staphylococcus is the most accused germ. Throughout this study, we report the case of pyomyositis revealed by a toxic staphylococcal shock syndrome.

## Patient and observation

LA is an-8-year old who had an open trauma of the left elbow with pus 15 days before he consulted the pediatric emergency department for a generalized scarlatiniform erythema evolving towards flaking with a fever of 39^º^C. Clinical examination has found a drowsy child, tachycardia at 130 beats per minute, polypnea at 44 cycles per minute, hypotension at 90/56mmHg with an extended capillary refill time (CRT) which required his admission to intensive care initially. The biological assessment had shown an inflammatory syndrome: C reactive protein (CRP) at 175.85mg/L; to the blood count: leukocytosis at 37.270g/L with a neutrophil predominance at 26.540g/L, thrombocytopenia at 83G/L and a lymphocyte count at 4.83g/L. The blood ionogram identified hyponatremia at 122mmol/L, hypokalemia at 3.4mmol/L and hypo-albuminemia at 23.4g/L. Two blood cultures were performed with isolation of a methicillin-sensitive coagulase negative staphylococcus A. The patient received amoxicillin-clavulanic acid intravenously for 21 days combined with gentamycin for 48 hours. The evolution a week later was marked by the appearance of a painful inflammatory swelling associated with spasticity of both arms and legs with functional impotence of the two lower limbs and a walk on tiptoes. The patient was transferred to our department after a stay in intensive care for etiological assessment.

**Figure 1:**
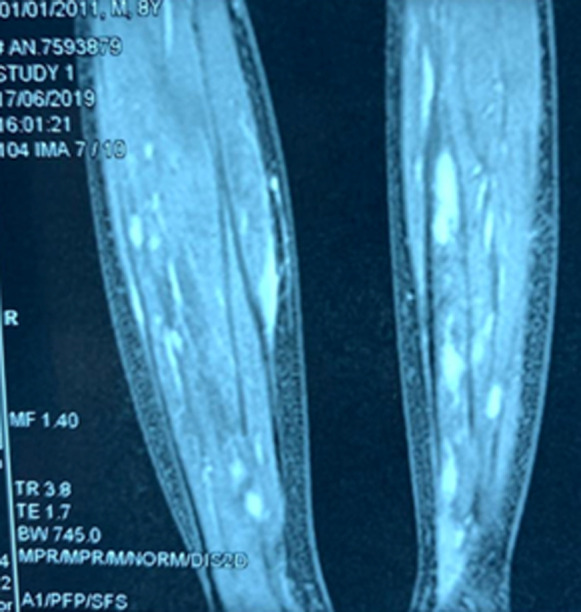
multiple abscess of the posterior compartment of both legs in T2 hyperintense signal

An X-ray of the pelvis and both elbows were done which were found to be normal with a creatine phosphokinase (CPK) level at 37IU/L. An ultrasound of 4 limbs was performed objectifying abscesses of soft tissues, magnetic resonance imaging (MRI) of the two legs revealed the presence of multiple multiloculated formations with T2 hyperintense signal on the posterior and the right antero-external compartment of both legs measuring for the largest 200 X 9 X 8 mm (Figure 1), associated with T2 hyperintense signal and edematous calcaneus on short-TI inversion recovery (STIR) images suggesting bilateral pyomyositis. Indeed, the delay between first signs and diagnosis was 2 months. Intravenous antibiotic therapy was started with amoxicillin-clavulanic acid for 4 weeks then 15 days orally, for a total duration of 6 weeks, associated with motor physiotherapy. The patient had after 20 days of evolution a drainage of abscesses by the team of orthopedic surgeons of our university hospital, however, the puncture was white; complemented by an incision which did not objectify pus with a sterile bacteriological study. Fever disappeared after 72 hours; the child gradually resumed walking during his hospitalization with a complete regression of the symptomatology after 6 weeks. Regarding the search for an underlying cause, the HIV serology was negative, the fasting blood sugar was 0.92g/dL, the lactate - ammonia level were normal and the dihydrorhodamine 123 test (DHR) for a chronic septic granulomatosis was normal. However, the assessment of immunoglobulins, lymphocyte subpopulations and vaccine antibodies was not carried out.

## Discussion

Pyomyositis or tropical myositis is a suppurative disease of the striated muscle originally described by Scriba in Japan [1]. The clinical symptomatology is insidious and not very specific, associating a fever, pain with inflammation of the involved location as well as general signs of sepsis [1,2]. In rare cases the presentation is that of a toxic shock syndrome, this was the case in our patient [3]. Indeed, it is not a very well-known disease simulating several pathologies which explains the diagnostic delay with an average delay described in the literature of 15 days (10-62 days) joining our study [1,4], hence the interest of recognizing pyomyositis in children in front of any painful and febrile lameness. Several authors have reported the concept of limb trauma as one of the factors favoring abscess formation with a predilection for the muscles of the lower limb and the small pelvis: psoas, external and internal obturators, quadriceps and adductor, which is also the case for our patient [2,5,6]. The increase in biological parameters (neutrophil leukocytosis, CRP) is the same regardless the topography [3], blood cultures are crucial for the identification of a germ but are positive in only half of the cases [7] accordingly the interest of bacteriological sampling of abscesses.

In our case, the two blood cultures were positive with isolation of *Staphylococcus aureus* (SA) which remains the most involved germ in the literature with a rate of 60% [2], but other germs can be responsible for pyomyositis such as streptococcus or anaerobes [8,9]. The role of radiological assessment in the diagnosis of pyomyositis is essential. X-rays allow eliminating certain differential diagnoses such as osteomyelitis or arthritis, ultrasound is sensitive and keeps its place in the drainage of abscesses and MRI remains the radiological examination of choice given its sensitivity and specificity; it allows the diagnosis to be made at an earlier stage [10]. Concerning the treatment of pyomyositis, it is not codified; the empirical antibiotic therapy of choice in the literature remains penicillin M intravenously for ten days since the main germ is the SA sensitive to methicillin with oral relay adapted to the antibiogram for a total duration of 4 to 6 weeks on average [5,7], for our patient we prescribed amoxicillin-clavulanic acid [4,6] considering the necrosis complication encountered with penicillin M during many experiences in our department and also that amoxicillin-clavulanic acid is active on staphylococcus and provides a good clinical improvement.

## Conclusion

Pyomyositis is an unrecognized and nonspecific disease that should always be kept in mind in front of clinical triad of fever, pain and lameness. Blood cultures and abscess samples can most often isolate a germ, but MRI remains the benchmark for positive diagnosis.
